# ESC Heart Failure increases its impact factor

**DOI:** 10.1002/ehf2.13069

**Published:** 2020-10-28

**Authors:** Markus S. Anker, Zoltán Papp, Gábor Földes, Stephan von Haehling

**Affiliations:** ^1^ Division of Cardiology and Metabolism, Department of Cardiology (CVK) Charité University Medicine Berlin Berlin Germany; ^2^ Berlin Institute of Health Center for Regenerative Therapies (BCRT) Berlin Germany; ^3^ DZHK (German Centre for Cardiovascular Research), partner site Berlin Berlin Germany; ^4^ Department of Cardiology (CBF) Charité University Medicine Berlin Berlin Germany; ^5^ Division of Clinical Physiology, Department of Cardiology, Faculty of Medicine University of Debrecen Debrecen Hungary; ^6^ HAS‐UD Vascular Biology and Myocardial Pathophysiology Research Group Hungarian Academy of Sciences Budapest Hungary; ^7^ National Heart and Lung Institute Imperial College London London UK; ^8^ Heart and Vascular Center Semmelweis University Budapest Hungary; ^9^ Department of Cardiology and Pneumology University Medical Center Göttingen Göttingen Germany; ^10^ DZHK (German Centre for Cardiovascular Research), partner site Göttingen Göttingen Germany

ESC Heart Failure (ESC‐HF) is an open access journal focused on advancing the understanding of heart failure since 2014. It is one of two journals of the Heart Failure Association of the European Society of Cardiology (European Journal of Heart Failure & ESC Heart Failure). ESC‐HF is published by Wiley as its publishing house. The journal's main topics include basic and translational research questions regarding the pathophysiology, electrophysiology, and biology of heart failure; clinical questions regarding the prevention, diagnosis, understanding, and therapy of heart failure; and epidemiological questions regarding the incidence, prevalence, prevention, distribution, and risk factors for heart failure in different populations. The workload regarding submitted journals is distributed between Prof. Stefan D. Anker (Berlin, Germany), the editor‐in‐chief, and the three deputy editors Prof. Stephan von Haehling (Göttingen, Germany) for the clinical section, Prof. Zoltán Papp (Budapest, Hungary) for the basic science section, and Dr Gábor Földes (London, UK, and Budapest, Hungary) for translational research. However, the journal would not be feasible without the help of the many authors and reviewers, editorial board members, associate editors, and especially the editorial office with Monika Diek, Anja Janssen, and Corinna Denecke. This journal relies on all these people to work together, towards the same goal of better understanding heart failure and its treatment.

While in 2014, new journal issues were published every 6 months, today every 2 months a new issue is published. Thereby, the number of articles steadily increased over the years—2014, 16; 2015 and 2016, both 41; 2017, 95; 2018, 131; and 2019, 149. This is a ninefold increase of published articles from 2014 until 2019. At the time of this writing in September 2020, the number of submissions in 2020 has reached 926. The journal attracts publications from many different nations. The list of countries where most of the articles originate includes Germany, the United States, Japan, England, Italy, Netherland, Spain, Sweden, France, and Australia.

In Web of Science,^1^ESC‐HF is currently ranked number 44 of 138 journals in the ‘cardiac & cardiovascular systems’ section. The top 10 best cited papers ever published in ESC‐HF (*Table* *1*) so far on average have already been cited 39 times. These top 10 papers included five original articles, two reviews, and even three editorials, showing that ESC‐HF does not only publish original articles but also state‐of‐the‐art reviews and editorials. This is confirmed when looking at the top 20 papers published in 2017–2019 (*Tables* *2*, *3*, *4*). In 2017, this included 15 original articles, three reviews, and two editorials; in 2018, 14 original articles and six reviews; and in 2019, 15 original articles, four reviews, and one editorial. Sixteen papers from 2017 and 2018 have been already cited ≥20 times, and even of the 2019 papers, three papers have already been cited ≥10 times.

**TABLE 1:** ESC Heart Failure top 10 cited articles of all time

Rank	Authors	Title	Article type	Times cited
1	Springer, J	Muscle wasting and sarcopenia in heart failure and beyond: update 2017^2^	Review	57
2	Luedde, M	Heart failure is associated with depletion of core intestinal microbiota^3^	Article	48
3	Jujo, K	Randomized pilot trial comparing tolvaptan with furosemide on renal and neurohumoral effects in acute heart failure^4^	Article	43
4	Lam, CSP	Heart failure in Southeast Asia: facts and numbers^5^	Editorial	41
5	Riley, JP	Palliative care in heart failure: facts and numbers^6^	Editorial	38
6	Konishi, M	Heart failure epidemiology and novel treatments in Japan: facts and numbers^7^	Editorial	36
7	Saitoh, M	Anorexia, functional capacity, and clinical outcome in patients with chronic heart failure: results from the Studies Investigating Co‐morbidities Aggravating Heart Failure (SICA‐HF)^8^	Article	35
8	Nagarajan, V	Obesity paradox in heart failure: a heavy matter^9^	Review	34
9	Mustroph, J	Empagliflozin reduces Ca/calmodulin‐dependent kinase II activity in isolated ventricular cardiomyocytes^10^	Article	30
10	Haghikia, A	Prognostic implication of right ventricular involvement in peripartum cardiomyopathy: a cardiovascular magnetic resonance study^11^	Article	29

**TABLE 2:** ESC Heart Failure top 20 cited articles published 2019

Rank	Authors	Title	Article type	Times cited
1	Vallabhajosyula, S	Sex disparities in acute kidney injury complicating acute myocardial infarction with cardiogenic shock^12^	Article	13
2	Nishi, I	Geriatric nutritional risk index predicts all‐cause deaths in heart failure with preserved ejection fraction^13^	Article	13
3	Linde, C	Serum potassium and clinical outcomes in heart failure patients: results of risk calculations in 21 334 patients in the UK^14^	Article	10
4	Veenis, JF	Design and rationale of haemodynamic guidance with CardioMEMS in patients with a left ventricular assist device: the HEMO‐VAD pilot study^15^	Article	9
5	Zabel, M	Rationale and design of the EU‐CERT‐ICD prospective study: comparative effectiveness of prophylactic ICD implantation^16^	Article	9
6	Grossekettler, L	Peritoneal dialysis as therapeutic option in heart failure patients^17^	Article	8
7	Lan, NSR	The effects of sodium‐glucose cotransporter 2 inhibitors on left ventricular function: current evidence and future directions^18^	Review	7
8	Chapman, B	Clinical profiles in acute heart failure: an urgent need for a new approach^19^	Review	7
9	Okonko, DO	Effect of ferric carboxymaltose on calculated plasma volume status and clinical congestion: a FAIR‐HF substudy^20^	Article	6
10	Ahmad, S	The functional consequences of sodium channel Na(V)1.8 in human left ventricular hypertrophy^21^	Article	6
11	Belyavskiy, E	Diastolic stress test echocardiography in patients with suspected heart failure with preserved ejection fraction: a pilot study^22^	Article	6
12	Gallagher, AM	Assessing health‐related quality of life in heart failure patients attending an outpatient clinic: a pragmatic approach^23^	Article	6
13	Polzl, G	Repetitive levosimendan infusions for patients with advanced chronic heart failure in the vulnerable post‐discharge period^24^	Article	6
14	Brownrigg, J	Diagnostic performance of imaging investigations in detecting and differentiating cardiac amyloidosis: a systematic review and meta‐analysis^25^	Review	5
15	Hagendorff, A	Echocardiographic assessment of functional mitral regurgitation: opening Pandora's box?^26^	Editorial	5
16	Korosoglou, G	Strain‐encoded magnetic resonance: a method for the assessment of myocardial deformation^27^	Review	5
17	Ibrahim, NE	Heart failure with mid‐range ejection fraction: characterization of patients from the PINNACLE Registry (R)^28^	Article	5
18	Shiga, T	Clinical characteristics of hospitalized heart failure patients with preserved, mid‐range, and reduced ejection fractions in Japan^29^	Article	5
19	Capucci, A	Preliminary experience with the multisensor HeartLogic algorithm for heart failure monitoring: a retrospective case series report^30^	Article	5
20	Creber, RM	Use of the Palliative Performance Scale to estimate survival among home hospice patients with heart failure^31^	Article	5

**TABLE 3:** ESC Heart Failure top 20 cited articles published 2018

Rank	Authors	Title	Article type	Times cited
1	Mustroph, J	Empagliflozin reduces Ca/calmodulin‐dependent kinase II activity in isolated ventricular cardiomyocytes^10^	Article	30
2	Cattadori, G	Exercise and heart failure: an update^32^	Review	26
3	Suzuki, T	Skeletal muscle wasting in chronic heart failure^33^	Review	25
4	Lauritsen, J	Characteristics and long‐term prognosis of patients with heart failure and mid‐range ejection fraction compared with reduced and preserved ejection fraction: a systematic review and meta‐analysis^34^	Review	23
5	Martens, P	Insights into implementation of sacubitril/valsartan into clinical practice^35^	Article	23
6	Pascual‐Figal, D	Rationale and design of TRANSITION: a randomized trial of pre‐discharge vs. post‐discharge initiation of sacubitril/valsartan^36^	Article	21
7	Seropian, IM	Neutrophil‐to‐lymphocyte ratio and platelet‐to‐lymphocyte ratio as predictors of survival after heart transplantation^37^	Article	21
8	Pitt, B	Evaluation of an individualized dose titration regimen of patiromer to prevent hyperkalaemia in patients with heart failure and chronic kidney disease^38^	Article	20
9	Norberg, H	Eligibility of sacubitril‐valsartan in a real‐world heart failure population: a community‐based single‐centre study^39^	Article	20
10	Pitt, B; Bakris	Long‐term effects of patiromer for hyperkalaemia treatment in patients with mild heart failure and diabetic nephropathy on angiotensin‐converting enzymes/angiotensin receptor blockers: results from AMETHYST‐DN^40^	Article	19
11	Lam, CSP	Iron deficiency in chronic heart failure: case‐based practical guidance^41^	Review	18
12	Ohman, J	Focused echocardiography and lung ultrasound protocol for guiding treatment in acute heart failure^42^	Article	18
13	Pappalardo, F	Full percutaneous biventricular support with two Impella pumps: the Bi‐Pella approach^43^	Article	17
14	Yoshihisa, A	Liver fibrosis score predicts mortality in heart failure patients with preserved ejection fraction^44^	Article	17
15	Smedema, JP	Right ventricular involvement and the extent of left ventricular enhancement with magnetic resonance predict adverse outcome in pulmonary sarcoidosis^45^	Article	17
16	Mints, YY	Features of atrial fibrillation in wild‐type transthyretin cardiac amyloidosis: a systematic review and clinical experience^46^	Review	16
17	Lavall, D	Mitral valve interventions in heart failure^47^	Review	15
18	Buckley, LF	Low NT‐proBNP levels in overweight and obese patients do not rule out a diagnosis of heart failure with preserved ejection fraction^48^	Article	15
19	Sawano, M	Performance of the MAGGIC heart failure risk score and its modification with the addition of discharge natriuretic peptides^49^	Article	14
20	Ferreira, JP	Rationale of the FIBROTARGETS study designed to identify novel biomarkers of myocardial fibrosis^50^	Article	14

**TABLE 4:** ESC Heart Failure top 20 cited articles published 2017

Rank	Authors	Title	Article type	Times cited
1	Springer, J	Muscle wasting and sarcopenia in heart failure and beyond: update 2017^2^	Review	57
2	Luedde, M	Heart failure is associated with depletion of core intestinal microbiota^3^	Article	48
3	Riley, JP	Palliative care in heart failure: facts and numbers^6^	Editorial	38
4	Saitoh, M	Anorexia, functional capacity, and clinical outcome in patients with chronic heart failure: results from the Studies Investigating Co‐morbidities Aggravating Heart Failure (SICA‐HF)^8^	Article	35
5	Barkhudaryan, A	Cardiac muscle wasting in individuals with cancer cachexia^51^	Article	26
6	Khan, MS	Renin‐angiotensin blockade in heart failure with preserved ejection fraction: a systematic review and meta‐analysis^52^	Review	21
7	Delepaul, B	Who are patients classified within the new terminology of heart failure from the 2016 ESC guidelines?^53^	Article	21
8	Morishita, T	Association between matrix metalloproteinase‐9 and worsening heart failure events in patients with chronic heart failure^54^	Article	19
9	Cohen‐Solal, A	Beta blocker dose and markers of sympathetic activation in heart failure patients: interrelationships and prognostic significance^55^	Article	18
10	Ancion, A	Serum albumin level and hospital mortality in acute non‐ischemic heart failure^56^	Article	18
11	Mockel, M	The role of procalcitonin in acute heart failure patients^57^	Review	17
12	Theidel, U	Budget impact of intravenous iron therapy with ferric carboxymaltose in patients with chronic heart failure and iron deficiency in Germany^58^	Article	17
13	Alma, LJ	Shared biomarkers between female diastolic heart failure and pre‐eclampsia: a systematic review and meta‐analysis^59^	Review	17
14	Buggey, J	Left ventricular global longitudinal strain in patients with heart failure with preserved ejection fraction: outcomes following an acute heart failure hospitalization^60^	Article	16
15	Lancellotti, P	Protocol update and preliminary results of EACVI/HFA Cardiac Oncology Toxicity (COT) Registry of the European Society of Cardiology^61^	Article	16
16	Amin, A	On admission serum sodium and uric acid levels predict 30 day rehospitalization or death in patients with acute decompensated heart failure^62^	Article	16
17	Jain, A	The renal‐cardiac connection in subjects with preserved ejection fraction: a population based study^63^	Article	15
18	Toma, M	Differentiating heart failure phenotypes using sex‐specific transcriptomic and proteomic biomarker panels^64^	Article	15
19	Silvetti, S	Rehospitalization after intermittent levosimendan treatment in advanced heart failure patients: a meta‐analysis of randomized trials^65^	Article	14
20	Jaarsma, T	Sexual function of patients with heart failure: facts and numbers^66^	Editorial	14

In 2019, ESC‐HF articles were altogether cited 1276 times.^1^ Of these citations, 835 (65%) were to papers published in 2017/2018. ESC‐HF published 214 citable articles in 2017 and 2018. This is important to know, because the 2019 Thomson Scientific journal impact factor of each journal (which is believed the most important rating tool for journals in Europe and the USA) was calculated by dividing the 2019 citations to articles published in 2017 and 2018 by the number of citable articles in 2017 and 2018. Hence, the 2019 journal impact factor of ESC‐HF is 3.902 (835/214).^1^ Considering that the 2018 impact factor of ESC‐HF was 3.407, this is an increase by 15% in just 1 year, which we think is great success. This shows that the hard work of all researchers, editors, associates, and reviewers that has been put into the articles published in the last years in ESC‐HF is being recognized by the scientific community. We want to continue this path in the future and thank all people that are involved in ESC‐HF, helping to publish scientific papers, with the goal to improve the understanding, prevention, and treatment of heart failure.

## Conflict of interest

None declared.
